# The role of IL-17 family cytokines in cardiac fibrosis

**DOI:** 10.3389/fcvm.2024.1470362

**Published:** 2024-10-22

**Authors:** Liqing Huang

**Affiliations:** Three Gorges University Hospital of Traditional Chinese Medicine & Yichang Hospital of Traditional Chinese Medicine, Yichang, China

**Keywords:** IL-17, cardiac fibrosis, fibroblast, myofibroblast, extracellular matrix

## Abstract

Myocardial fibrosis is a common pathological feature in various cardiovascular diseases including myocardial infarction, heart failure, and myocarditis. Generally, persistent myocardial fibrosis correlates with poor prognosis and ranks among the leading causes of death globally. Currently, there is no effective treatment for myocardial fibrosis, partly due to its unclear pathogenic mechanism. Increasing studies have shown IL-17 family cytokines are strongly associated with the initiation and propagation of myocardial fibrosis. This review summarizes the expression, action, and signal transduction mechanisms of IL-17, focusing on its role in fibrosis associated with cardiovascular diseases such as myocardial infarction, heart failure, hypertension, diabetes, and myocarditis. It also discusses its potential as a therapeutic target, offering new insights for the clinical treatment of myocardial fibrosis.

## 1 Introduction

In recent years, the prevalence of fibrosis related diseases has markedly increased, emerging as a significant public health concern (1). Approximately 45% of disease related deaths are estimated to be linked to fibrosis (1, 2). Fibrosis contributes significantly to organ dysfunction across various diseases (3). Myocardial fibrosis refers to the excessive deposition of extracellular matrix (ECM) in the myocardial interstitium, a common pathophysiological feature associated with numerous heart diseases (3, 4). Myocardial fibrosis can signify either a reparative process or sustained injury. For instance, in myocardial infarction, early replacement fibrosis helps to maintain the intact structure of the heart. Conversely, in many cardiac pathologies like hypertensive heart disease, aortic stenosis, and diabetic cardiomyopathy, myocardial fibrosis represents a chronic, ongoing injury process (3).

According to homology analysis, the Interleukin 17 (IL-17) family includes IL-17A, IL-17B, IL-17C, IL-17D, IL-17E, and IL-17F (5, 6). These IL-17 family members are primarily derived from immune or non-hematopoietic cells and play diverse roles in immune responses, inflammation, and fibrotic diseases (6–10). Studies have demonstrated that in myocardial infarction, heart failure, diabetes, myocarditis, and other conditions associated with myocardial fibrosis, the expression of IL-17 in the heart or serum is significantly increased. Growing evidence indicates that IL-17 promotes the activation, proliferation, migration, and secretion of myocardial fibroblasts, playing a crucial role in regulating myocardial fibrosis. Therefore, this article aims to explore the roles and mechanisms of IL-17 family members in myocardial fibrosis associated with various cardiovascular diseases, offering new insights for their diagnosis and treatment.

## 2 Characteristics of the IL-17 family cytokines

IL-17 was initially discovered in 1993 (5), followed by the identification of other IL-17 family members: IL-17B, IL-17C, IL-17D, IL-17E, and IL-17F (6). Among them, IL-17A is the most extensively studied. The IL-17A gene is located on human chromosome 6p12. Human IL-17A is a 35kDa homodimeric protein (11, 12). IL-17F exhibits the highest sequence homology to IL-17A (55%), followed by IL-17B (29%), IL-17D (25%), IL-17C (23%), and IL-17E (16%) (6, 13). IL-17 is primarily produced by Th17 cell subsets, with additional contributions from γδT cells and NK cells (7).

The amino acid homology of IL-17 family members varies among humans, mice, and rats. Mouse IL-17A is a 21kDa glycoprotein composed of 147 amino acid residues, exhibiting 63% amino acid homology with human IL-17A (155 amino acids) and 58% homology with rat IL-17A. Both mouse and human IL-17A proteins are secreted as disulfide-linked homodimers (14). In addition, other members of the IL-17 family share 70%–90% amino acid sequence consistency in mice and humans. Specifically, in mice, IL-17B, IL-17C, IL-17D, IL-17E, and IL-17F share 88%, 83%, 78%, 81%, and 77% amino acid sequence similarity, respectively, with their human counterparts (14–17). Therefore, these differences suggest that the function and expression patterns of IL-17 may vary by species, an important consideration in studies of the IL-17 family.

The Interleukin-17 receptor (IL-17R) family comprises five receptor subunits: IL-17RA, IL-17RB, IL-17RC, IL-17RD, and IL-17RE. IL-17RA acts as a co-receptor subunit, forming heterodimer receptor complexes with other receptor subunits (14). IL-17 family members bind to their respective IL-17 receptor complexes to mediate downstream signaling pathways and participate in immunity, inflammation, fibrosis (6, 8–10). Specifically, IL-17A or IL-17F binding to the IL17RA/RC complex, IL-17B or IL-17E binding to the IL-17RA/RB complex, and IL-17C binding to the IL-17RA/RE complex (14). Recent studies have shown that IL-17D can bind CD93 to regulate intestinal homeostasis (15), but the receptor subunits specific to IL-17RD dimers remain incompletely defined.

**Figure 1:**
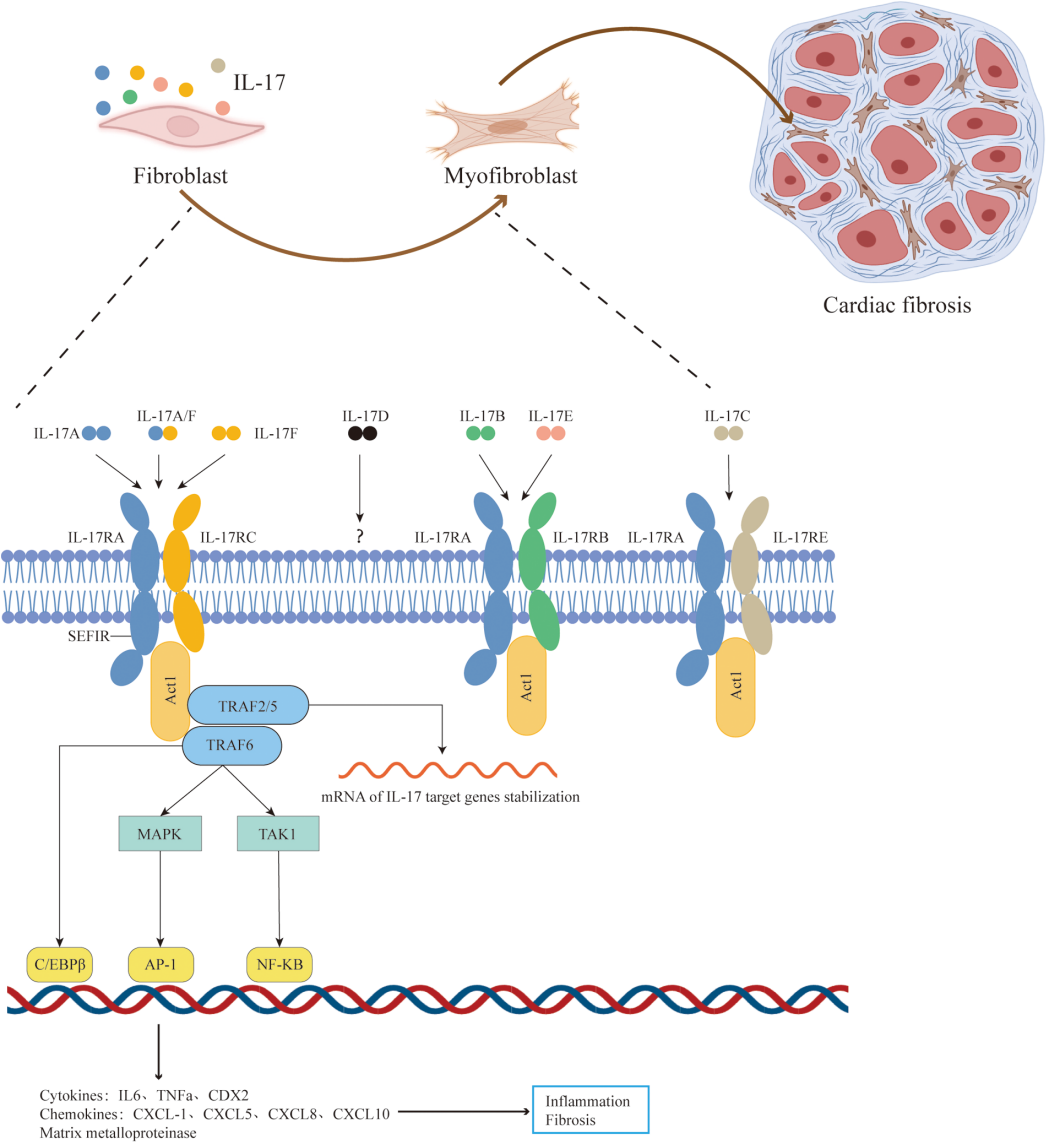
Pathophysiologic roles of IL-17 signaling in cardiac fibrosis. The IL-17 family cytokines include IL-17A, IL-17B, IL-17C, IL-17D, IL-17E, and IL-17F. The IL-17A/IL-17F mediated signaling pathway has been extensively studied. After IL-17A/IL-17F binds to the IL17RA/RC receptor complex, the receptor complex recruits the signal transduction adaptor protein Act1 through its SEFIR domain.Act1 serves as the crucial molecule for all known downstream signaling pathways of IL-17A. Act1 rapidly recruits and ubiquitinates TRAF6, activating various downstream pathways, including: the TRAF6/TAK1/NF-κB pathway, TRAF6/MAPK/AP-1 pathway, and Act1/TRAF6/CREB pathway, which induce transcription of IL-17A target genes. Furthermore, Act1 promotes mRNA stabilization post-transcription by recruiting TRAF2 and TRAF5. Ultimately, IL-17 induces the expression of pro-inflammatory and pro-fibrosis molecules and stimulate activation, proliferation, migration, and secretion of myocardial fibroblasts, thereby aggravating cardial fibrosis. Adapted with permission from “Cell Types”, by BioRender.com (2024), licensed under Academic License. Created in BioRender. T4, V. (2022).

The IL17RA/RC receptor complex mediated signaling pathway has been extensively studied (16–18). After IL-17A/IL-17F binds to the IL17RA/RC receptor complex, the receptor complex recruits the signal transduction adaptor protein Act1 through its SEFIR domain. Act1 serves as the crucial molecule for all known downstream signaling pathways of IL-17A. Act1 rapidly recruits and ubiquitinates TNF receptor-associated factor (TRAF)6, activating various downstream pathways, including: the TRAF6/TAK1/NF-κB pathway, TRAF6/MAPK/AP-1 pathway, and Act1/TRAF6/CREB pathway, which induce transcription of IL-17A target genes. Furthermore, Act1 promotes mRNA stabilization post-transcription by recruiting TRAF2 and TRAF5. Ultimately, IL-17 induces the expression of cytokines (IL-6, GM-CSF, TNF-α), chemokines (CXCL1, CXCL2, CCL20), and matrix metalloproteinases (MMPs), promoting inflammation and fibrosis (14, 19–21) (Figure 1). Additionally, IL-17RB, IL-17RD, and IL-17RE also contain SEFIR domains, suggesting a potentially similar activation mechanism (16, 22).

## 3 IL-17 family cytokines in fibrotic diseases

The fibrotic response consists of four stages: organ damage, effector cell activation, increased production of extracellular matrix (ECM), and dynamic deposition of connective tissue. Among these, the activation of effector cells is crucial, as they secrete cytokines, chemokines, and growth factors that establish the inflammatory foundation. Chronic and persistent inflammation serves as a key determinant of fibrosis. Specifically, IL-17, a pro-inflammatory cytokine, has been implicated in various inflammatory diseases. However, its role in the fibrosis of different organs remains complex (23). This section will briefly discuss the contributions of IL-17 family members to fibrotic diseases.

### 3.1 IL-17A

IL-17A is the most extensively studied member of the IL-17 family. While most studies indicate that IL-17 promotes organ fibrosis, some research presents opposing conclusions. This discrepancy suggests that the role of IL-17A in organ fibrosis is complex and warrants further investigation. In models of pulmonary fibrosis induced by bleomycin (BLM) and silica, the administration of anti-IL-17A antibodies has been shown to reduce pulmonary fibrosis and extracellular matrix (ECM) deposition (24–26). Conversely, γδ T cells producing IL-17 have been found to protect against BLM-induced pulmonary fibrosis by limiting inflammation and promoting epithelial regeneration (27). Similarly, several studies demonstrate that the absence of IL-17 signaling contributes to atherogenic effects in ApoE−/− mice (28–30). However, another study indicates that IL-17 deficiency may exacerbate atherosclerotic lesions in the aortic arch of ApoE−/− mice (31). This complexity in the role of IL-17A in fibrotic diseases is also evident in skin fibrosis (32, 33), kidney fibrosis (34, 35), and intestinal fibrosis (36, 37).

### 3.2 IL-17B

Research on the role of IL-17B in fibrotic diseases is limited, and its function remains largely unexplained. Current studies indicate that IL-17B is associated with the occurrence and progression of pulmonary fibrosis and systemic sclerosis. Notably, in systemic sclerosis, there is no statistically significant difference in serum IL-17A concentrations between systemic sclerosis(SSc) patients and controls. However, serum levels of IL-17B and IL-17E are elevated in patients with systemic sclerosis compared to controls (38). Additionally, in a mouse model of bleomycin-induced pulmonary fibrosis, IL-17B deficiency has been shown to slow the progression of fibrosis (39).

### 3.3 IL-17C

IL-17C is expressed in CD4T cells, dendritic cells, macrophages, and epithelial cells, where it promotes inflammation during antibacterial processes (40, 41). Its role in fibrosis progression is primarily focused on pulmonary fibrosis. In models of lung inflammation induced by Haemophilus influenzae and cigarette smoke, IL-17C is implicated in promoting lung neutrophil inflammation and contributing to pulmonary fibrotic injury (42).

### 3.4 IL-17D

IL-17D expression is more limited and is primarily detected in B lymphocytes and CD4T cells. It particularly acts on endothelial cells, regulating their secretion of pro-inflammatory factors (43). Clinical data indicate that in a cohort of 2,032 heart failure patients, higher plasma IL-17D concentrations are significantly associated with atrial fibrillation, increased levels of plasma N-terminal brain natriuretic peptide precursor, and poorer prognosis (44). Therefore, IL-17D may mediate pro-inflammatory and profibrotic effects.

### 3.5 IL-17E

IL-17E is closely associated with pulmonary fibrosis and SSc. It is secreted by Th2 cells, epithelial cells, endothelial cells, T cells, alveolar macrophages, dendritic cells (DC), eosinophils, and basophils (45). Hams found increased levels of IL-17E in lung tissue from patients with idiopathic pulmonary fibrosis, particularly in alveolar epithelial cells and lung fibroblasts (46). Further studies have shown that IL-17E promotes pulmonary fibrosis by directly activating lung fibroblasts and regulating the epithelial-mesenchymal transition (46, 47). Additionally, serum levels of IL-17RB and IL-17RC are elevated in patients with systemic sclerosis compared to healthy controls (48). In the presence of IL-17E and IL-17F, the production of IL-6, MMP-1, and MCP-1 by human healthy skin fibroblasts is enhanced in a dose-dependent manner (49).

### 3.6 IL-17F

IL-17F and IL-17A share a high degree of sequence homology, and the cells that produce IL-17F are the same as those that release IL-17A. However, the roles of IL-17F and IL-17A in fibrotic diseases differ. IL-17F can stimulate the release of IL-6 and CXC chemokines from tracheal epithelial cells, inflammatory cells, endothelial cells, and fibroblasts (50). In patients with hepatitis C virus in the severe fibrotic stage, serum IL-17F concentrations are significantly elevated (51). Additionally, IL-17F promotes the mRNA expression of collagen I in fibrocytes (52). In contrast, another study found that treatment with anti-IL-17F antibodies had no effect on chronic intestinal inflammation and fibrosis (53). Similarly, in an angiotensin II (Ang II)-induced mouse hypertensive model, the administration of anti-IL-17A or anti-IL-17RA antibodies reduced blood pressure by 30 mmHg and lowered renal fibrosis, compared to control IgG antibodies. However, anti-IL-17F treatment did not significantly affect blood pressure and renal fibrosis (54).

## 4 Cardiac fibrosis

Myocardial fibrosis refers to excessive deposition of ECM in the myocardial interstitium, which is a common pathological feature in cardiovascular diseases such as ischemic cardiomyopathy, dilated cardiomyopathy, and heart failure. According to the different patterns of fiber deposition, cardiac fibrosis can be divided into three categories: (1) Replacement fibrosis: necrotic cardiomyocytes are replaced by collagen fibers. (2) Interstitial fibrosis: In the absence of significant loss of cardiomyocytes, ECM accumulates in the myocardial interstitium. (3) Perivascular fibrosis: Collagen fibers accumulate in the outer membrane of myocardial microvessels. Replacement fibrosis often serves a reparative and protective role, whereas interstitial and perivascular fibrosis typically result from chronic inflammation or fibrotic stimuli, representing ongoing primary damage processes (3). Excessive ECM deposition under various pathological conditions can lead to myocardial stiffness, ultimately causing myocardial systolic and diastolic dysfunction (55).

Activated fibroblasts are the primary cells responsible for producing ECM and promoting myocardial fibrosis (3). These fibroblasts can transform into secretory myofibroblasts under the stimulation of various cytokines and growth factors, thereby inducing myocardial fibrosis (56, 57). Additionally, endothelial cells may directly contribute to myocardial fibrosis through endothelial-mesenchymal transition (58, 59). IL-17 plays a crucial role in this process by inducing activation, proliferation, migration, and secretion of myocardial fibroblasts (60, 61). Researches indicate that primary human cardiac fibroblasts express both IL-17RA and IL-17RC, along with IL-17A (61–63), suggesting the presence of autocrine and paracrine IL-17 signaling in these cells. In isolated human primary myocardial fibroblasts, IL-17 was found to stimulate MMP-1 expression through the p38 MAPK and ERK1/2 pathways (61). Similarly, in primary mouse cardiac fibroblasts, IL-17 was shown to enhance proliferation and migration of cardiac fibroblasts via the Akt/miR-101/MKP-1-dependent activation of p38 MAPK and ERK1/2 pathways (60).

## 5 IL-17 family cytokines in conditions associated with cardiac fibrosis

### 5.1 Fibrosis in myocardial infarction

Myocardial infarction results in the death of numerous cardiomyocytes, triggering an inflammatory response that leads to myocardial fibrosis. Early fibrosis at the infarction site is crucial for maintaining heart structure and has reparative benefits. However, excessive myocardial fibrosis can impair both systolic and diastolic heart function, ultimately causing heart failure. Therefore, strict regulation of inflammation and fibrosis post-myocardial infarction is essential to prevent excessive fibrosis while preserving heart structural integrity (3, 64).

A study of 981 patients with myocardial infarction found that high serum levels of IL-17(commonly refers to as IL-17A) were associated with a lower risk of death and recurrent myocardial infarction (65). In contrast, animal studies have demonstrated the opposite effect. In a mouse model of myocardial infarction induced by coronary artery ligation, a notable increase in IL-17A and IL-17RA expression was observed in the heart. IL-17RA was highly expressed in cardiomyocytes, fibroblasts, and macrophages. Flow cytometry analysis indicated that IL-17A was predominantly derived from γδT cells (8). *In vivo*, the administration of recombinant mouse IL-17A promoted myocardial fibrosis and ventricular remodeling 28 days after myocardial infarction in mice, compared to the myocardial infarction group (66). Additionally, another study revealed that IL-17A knockout mice exhibited a 30% increase in survival rate at day 28 compared to mice with a myocardial infarction model. Importantly, pathological staining analysis revealed a significant reduction in the non-infarct fibrotic area, whereas no reduction in the infarct fibrotic area in IL-17A knockout mice. PCR analysis showed that the expression of myocardiac fibrosis gene (MMP1, MMP3, MMP9, and C-C motif chemokine ligand 2 (CCL2) in IL-17A knockout mice did not significantly decrease on day 2 but showed a significant decrease on day 7. Meanwhile, cardiac ultrasound indicated that left ventricular dilation and systolic dysfunction were significantly alleviated in IL-17A knockout mice. These findings suggest that inhibition of IL-17A has no significant effect on early reparative fibrosis post-myocardial infarction, but can markedly inhibit excessive fibrosis post-myocardial infarction, thereby improving cardiac function and prognosis (8).

To further explore the mechanism of IL-17A, cardiomyocytes, myofibroblasts, and macrophages were isolated on the 7th day post-myocardial infarction. PCR analysis indicated that IL-17A knockout enhanced the survival of mouse cardiomyocytes, reduced the expression of pro-fibrosis molecules (CCL2, MMP3, and MMP9) in myocardial fibroblasts, and decreased the expression of pro-inflammatory molecules (TNF-α, IL-6, and IL-1β) in macrophages. *In vitro*, the expression of pro-inflammatory and pro-fibrosis molecules in fibroblasts increased by IL-17, and was inhibited by anti-IL-17A monoclonal antibody (8). Moreover, IL-17A has been shown to induce cardiomyocyte apoptosis via the p38-MAPK-p53-Bax signaling pathway (66).

In summary, the role of IL-17 in myocardial infarction in humans and animals has yielded conflicting conclusions. One possible explanation for this discrepancy may be the differences in sequence homology of IL-17 between humans and mice/rats. Additionally, the effects of IL-17 could be influenced by factors such as the type and duration of myocardial infarction, which warrant further investigation.

### 5.2 Fibrosis in the pressure-overloaded heart

Inflammation plays a crucial role in myocardial injury. Hypertension induces cardiac fibrosis characterized by low-grade inflammation. Persistent hypertension leads to cardiac remodeling, including myocardial hypertrophy and fibrosis, subsequently resulting in ventricular systolic and diastolic dysfunction (67). Several studies have revealed an association between the IL-17 and fibrosis in the pressure-overloaded heart (10, 68, 69). *In vitro*, IL-17A stimulated fibroblasts to produce pro-inflammatory factors such as IL-6 and TNF-α, and promoted myofibroblasts differentiation in an IL-6-dependent manner (10). *In vivo*, Angiotensin II (AngII) increased cardiac IL-17A mRNA expression in a time-dependent manner in a model of AngII-induced hypertensive myocardial injury. Compared with mice in early (1 week) and long-term (4 weeks) AngII-induced hypertension models, IL-17A knockout mice exhibited significantly reduction of myocardial fibrosis. Western Blot and immunohistochemical staining revealed the expression of myocardial α-smooth muscle actin (α-SMA) was significantly decreased, suggesting reduced myofibroblasts activation in IL-17A knockout mice (10). Additionally, in the spontaneously hypertensive rat model, specific targeting of IL-17RA therapy significantly improved myocardial fibrosis and cardiac function, reducing MMP-2, MMP-9, type I and III collagen expression (68). Moreover, in a hypertensive rat model induced by deoxycorticosterone acetate and high-salt diet, salt-corticosteroid receptor activation promotes myocardial inflammation and fibrosis through the Th17/Treg/IL-17 pathway, resulting in cardiac damage. Salocorticoid receptor antagonists can block Th17 cell activation and IL-17 signaling, thereby inhibiting myocardial inflammation and fibrosis (69). Furthermore, treatment with anti-IL-17 antibodies significantly decreased pro-inflammatory and pro-fibrotic factors (TGF-β1, OPN, NOX-2) and type I collagen expression in the heart and kidney (69).

In contrast, in a mouse model of hypertension induced by deoxycorticosterone acetate and angiotensin II, IL-17 knockout mice did not exhibit lower systolic blood pressure or improvements in target organ damage on days 4 and 14. Additionally, no significant differences were observed in heart weight and cardiac fibrosis compared to wild-type mice (70). Similarly, in a model of angiotensin II-induced myocardial injury, treatment with anti-IL-17A antibodies did not reduce myocardial hypertrophy or fibrosis (71).

In conclusion, the role of IL-17 in hypertension and hypertensive myocardial fibrosis remains uncertain. The differing conclusions may be attributed to variations in hypertension models or the duration of the studies. Generally, prolonged exposure to higher blood pressure levels is more likely to induce myocardial fibrosis. Additionally, IL-17 may play distinct roles at various stages of hypertensive myocardial fibrosis.

### 5.3 Fibrosis in diabetes

Diabetic cardiomyopathy stands as a leading cause of mortality among diabetic patients. The onset of diabetes-induced myocardial fibrosis crucially leads to both diastolic and systolic dysfunction in the heart, furthering the progression of diabetic cardiomyopathy (72). Numerous studies underscore IL-17's role in advancing diabetic myocardial fibrosis (72–74). Yanqing Qi et al. documented that the serum level of IL-17 was significantly elevated in the diabetic patients (72). Subsequently, in a mouse model of type 1 diabetes induced by three consecutive intravenous injections of streptomycin (50 mg/kg), IL-17 expression was significantly upregulated in both serum and heart tissue. Upon IL-17 knockout, diabetic mice exhibited substantially reduced myocardial fibrosis and improved cardiac systolic and diastolic functions (73). Moreover, research indicates that lncRNA-MIAT (Myocardial Infarction Associated Transcript, attenuates miR-214-3p mediated inhibition of IL-17 expression)-induced IL-17 elevation plays a pivotal role in diabetic myocardial fibrosis (72). Additionally, anthocyanins’ capacity to downregulate IL-17 expression has shown promising results in ameliorating diabetic myocardial fibrosis (74). Further investigation has illuminated IL-17's role in promoting collagen synthesis in myocardial fibroblasts via the lncRNA AK081284/TGF*β*1 signaling pathway (73).

### 5.4 Fibrosis in myocarditis

Myocarditis, an inflammatory myocardial disease resulting from both infectious and non-infectious insults, is a frequent precursor to Dilated Cardiomyopathy (DCM) and sudden cardiac death (75, 76). Approximately 30% of myocarditis cases progress to DCM, which is a significant cause of sudden death among young adults (75, 77, 78). Myocardial fibrosis stands out as a critical mechanism driving cardiac dysfunction progression in patients with myocarditis and cardiomyopathy (3).

Recent studies have highlighted IL-17 as a crucial mediator of cardiac remodeling and DCM following myocarditis (4, 78, 79). IL-17A expression was markedly increased in hearts of mice with autoimmune myocarditis (4, 79). Similarly, in a myocarditogenic peptide induced mouse autoimmune myocarditis model using complete Freund's adjuvant emulsion,significant Th17 cells infiltration into the myocardium and progressive left ventricular dilation were observed. Flow cytometry analysis indicated IL-17A specifically upregulated IL-6, TNF-α, and IL-1β, recruiting inflammatory cells to the heart. Mice treated with IL-17A knockout or IL-17A monoclonal antibody exhibited significantly reduced cardiac remodeling and myocardial fibrosis post-myocarditis, and absence of DCM (78). Lowering IL-17A levels also downregulated systemic TNF-α and IL-6 levels, decreasing subsequent left ventricular dilation and mortality in a Coxaxy virus B3-induced chronic myocarditis mouse model (80). Fibroblast plays a pivotal role in myocardial fibrosis (60, 61). Bioinformatics analysis identified the IL-17 signaling pathway is closely related to the pathogenesis of DCM (81). Further investigation demonstrated IL-17A's potential to induce type I and III collagen expression in fibroblasts via the PKCβ/ERK1/2/NF-κB pathway (4). Additionally, in a autoimmune myocarditis porcine model induced by myosin, IL-17 significantly increased MMP-2 and MMP-9 expression in cardiac fibroblasts through the OPG/RANK/RANKL pathway, thereby promoting post-myocarditis cardiac remodeling (82).

### 5.5 Fibrosis in heart failure

Heart failure is a leading cause of death and hospitalization worldwide. Cardiac fibrosis is a significant pathological manifestation of heart failure. In many cases, persistent myocardial fibrosis can lead to the onset and progression of heart failure. The severity of myocardial fibrosis is closely related to poor prognosis (3, 83).

Myocardial fibrosis can lead to diastolic dysfunction of the heart and plays an important role in heart failure with preserved ejection fraction (3). Clinical studies have shown that serum levels of IL-17 and IL-6 are significantly increased in patients with left ventricular diastolic dysfunction. Furthermore, logistic regression analysis indicated that the combined elevation of IL-17 and IL-6 is an independent predictor of poor prognosis in left ventricular diastolic dysfunction (84). Additionally, increased IL-17 levels are significantly associated with elevated fibrosis levels (9, 84). IL-17 can also induce the proliferation and secretion of cardiac fibroblasts (derived from human or mice) (8, 60, 61), making it a crucial molecule in promoting fibrosis in heart failure (84–87).

The imbalance of the helper T cell (Th17)/regulatory T cell (Treg) ratio is closely associated with chronic heart failure. In patients with heart failure, the Th17/Treg ratio is disrupted, with Th17 being significantly increased. Th17 promotes inflammation and fibrosis primarily through the secretion of IL-17 (86). In the rat model of chronic heart failure induced by aortic coarctation, Infusion of Th17 cells increased myocardial fibrosis and heart failure compared to the model group. Similarly, in the rabbit model of ischemic heart failure, intravenous infusion of IL-17(twice a week for 4 weeks) also aggravated myocardial fibrosis and heart failure. TUNEL staining and cleaved caspase-3 staining demonstrated that IL-17 promoted apoptosis of cardiomyocytes (86, 87). Furthermore, in the rat model of heart failure induced by isoproterenol, HE and Masson staining analysis revealed a significant reduction in myocardial fibrosis by the treatment with anti-IL-17 monoclonal antibody. Type I and III collagen fiber levels were also notably decreased (85). Collagen synthesis and degradation are regulated by MMPs and TIMPs, with an increased MMP/TIMP ratio promoting myocardial fibrosis (88–90). *In vitro*, IL-17 induced MMP-1 expression in cardiac fibroblasts, partly through the RANKL/OPG signaling pathway. Therefore, IL-17 may promote myocardial fibrosis via the RANKL/OPG signaling pathway and MMP/TIMP systems (85). In summary, IL-17 acts as a promoter of cardiac fibrosis in multifactorial heart failure, including sympathetic overactivation and ischemic heart failure, suggesting it as a significant therapeutic target for heart failure treatment.

IL-17 induces ventricular remodeling and ventricular arrhythmias in ischemic heart failure (87). Lysyl oxidase (LOX) is a copper-dependent extracellular enzyme, associated with myocardial fibrosis and cardiac dysfunction (86, 91). Research has demonstrated that IL-17 exacerbates myocardial fibrosis and heart failure by activating the ERK1/2-AP-1 pathway to induce LOX expression (86). Rhodiola crenulata has been found to inhibit IL-17 expression and its downstream target genes, thereby improving fibrosis and reducing apoptosis, as well as inhibiting ventricular arrhythmias (92). Moreover, multivariate Cox regression analysis revealed that elevated plasma IL-17D levels were associated with significantly increased risks of combined outcomes including atrial fibrillation, heart failure hospitalization, and all-cause mortality (44).

### 5.6 Fibrosis in heart transplantation

Heart transplantation is an effective treatment for end-stage heart failure, but chronic rejection remains a critical factor impacting long-term graft survival. Chronic rejection is characterized by myocardial hypertrophy, interstitial fibrosis, vascular occlusion, and progressive graft dysfunction (93). The intricate interplay among IL-17, TGF-β, IL-6, and CTGF plays a pivotal role in heart transplant rejection. Researches demonstrated that heart allografts in IL-17 knockout mice exhibited markedly reduced fibrosis and significantly prolonged graft survival compared to wild-type controls (94–96). Thus, IL-17 represents a promising therapeutic target for combating fibrosis associated with heart transplantation.

### 5.7 Others

In a rat model of aseptic pericarditis, IL-17A promotes the development of atrial fibrillation by inducing myocardial fibrosis (97). This effect is likely mediated through the stimulation of atrial fibrillation-related inflammatory cytokines such as IL-1β, IL-6, and TGF-β1 (60, 97). Additionally, elevated serum IL-17 levels was observed in patients with systemic lupus erythematosus-associated myocardial injury. Univariate logistic regression analysis indicated a correlation between serum IL-17 levels and myocardial injury in these patients (98). Furthermore, chemotherapeutic agents can induce various forms of cardiotoxicity, with sunitinib primarily associated with myocardial fibrosis. In a rat model of sunitinib-induced myocardial fibrosis, secukinumab (an IL-17A inhibitor) significantly attenuated myocardial fibrosis and improved left ventricular systolic and diastolic function. Additional investigations suggested that IL-17 might enhance fibrosis by modulating the OPG/RANK/RANKL axis (99).

## 6 Conclusion

In recent years, IL-17 has garnered increasing attention in chronic inflammation, organ fibrosis, and autoimmune diseases. IL-17 is predominantly secreted by immune or non-hematopoietic cells, mediating downstream gene expression through various signaling pathways. It directly or indirectly promotes cardiac fibroblast activation and regulates ECM production. Thus, IL-17 plays a crucial role in fibrotic processes associated with cardiovascular diseases such as myocardial infarction, heart failure, hypertension, diabetes, and myocarditis. Most current studies suggest that IL-17 can promote myocardial fibrosis, the anti-IL-17 biologic sukinzumab has been approved for the treatment of moderate to severe psoriasis (100). Interestingly, the role of IL-17 in post-infarction fibrosis and hypertensive myocardial fibrosis appears contradictory. This discrepancy may be related to the different stages and models of the disease. Furthermore, it is noteworthy that the mechanism of IL-17 action may be more complex than previously imagined, indicating that more in-depth studies are needed to explore its role in myocardial fibrosis, which could provide a foundation for precise targeted treatments for clinical myocardial fibrosis.
